# Identification of risk factors for venous thromboembolism and validation of the Khorana score in patients with advanced lung cancer: based on the multicenter, prospective Rising-VTE/NEJ037 study data

**DOI:** 10.1007/s10147-022-02257-y

**Published:** 2022-11-10

**Authors:** Yukari Tsubata, Keita Kawakado, Kosuke Hamai, Naoki Furuya, Toshihide Yokoyama, Ryota Saito, Atsushi Nakamura, Takeshi Masuda, Megumi Hamaguchi, Shoichi Kuyama, Ryoichi Honda, Tadashi Senoo, Masamoto Nakanishi, Takamasa Hotta, Masahiro Yamasaki, Nobuhisa Ishikawa, Kazunori Fujitaka, Tetsuya Kubota, Kunihiko Kobayashi, Takeshi Isobe

**Affiliations:** 1 Division of Medical Oncology and Respiratory Medicine, Department of Internal Medicine, Shimane University Faculty of Medicine, 89-1 Enya-Cho, Izumo, Shimane 693-8501 Japan; 2 Department of Respiratory Medicine, Hiroshima Prefectural Hospital, 1-5-54 Ujina-Kanda, Minami-ku, Hiroshima, 734-8530 Japan; 3 Division of Respiratory Medicine, Department of Internal Medicine, St. Marianna University School of Medicine, 2-16-1 Sugao, Miyamae-ku, Kawasaki, Kanagawa 216-8511 Japan; 4 Department of Respiratory Medicine, Kurashiki Central Hospital, 1-1-1, Miwa, Kurashiki, Okayama 710-8602 Japan; 5 Department of Respiratory Medicine, Tohoku University, 1-1 Seiryo-Machi, Aoba-ku, Sendai, Miyagi 980-8574 Japan; 6 Department of Pulmonary Medicine, Sendai Kousei Hospital, 4-15 Hirose-Machi, Aoba-ku, Sendai, Miyagi 980-0873 Japan; 7 Department of Respiratory Medicine, Hiroshima University Hospital, 1-2-3 Kasumi, Minami-ku, Hiroshima, 734-8553 Japan; 8 Department of Respiratory Medicine, Iwakuni Clinical Center, 1-1-1 Atago-Machi, Iwakuni, Yamaguchi 740-8510 Japan; 9 Department of Respiratory Medicine, Asahi General Hospital, I-1326 Asahi, Chiba, 289-2511 Japan; 10 Department of Respiratory Medicine, National Hospital Organization, Kure Medical Center, 3-1 Aoyamacho, Kure, Hiroshima 737-0023 Japan; 11 Department of Medical Oncology, Yamaguchi-Ube Medical Center, 685 Higashikawa, Ube, Yamaguchi 755-0241 Japan; 12 Department of Respiratory Disease, Hiroshima Red Cross Hospital and Atomic-Bomb Survivors Hospital, 1-9-6, Senda-Machi, Naka-ku, Hiroshima, 730-8619 Japan; 13 Department of Respiratory Medicine and Allergology, Kochi University Hospital, 185-1 Kohasu, Oko-Cho, Nankoku, Kochi 783-8505 Japan; 14 Department of Pulmonary Medicine, Saitama Medical University International Medical Center, 1397-1 Yamane, Hidaka, Saitama 350-1298 Japan

**Keywords:** Deep vein thrombosis, Pulmonary thromboembolism, Lung cancer, Khorana score, Prothrombin fragment 1 + 2

## Abstract

**Background:**

Management of cancer-associated venous thromboembolism (VTE) is essential in cancer treatment selection and prognosis. However, currently, no method exists for assessing VTE risk associated with advanced lung cancer. Therefore, we assessed VTE risk, including driver gene mutation, in advanced lung cancer and performed a Khorana score validation.

**Methods:**

The Rising-VTE/NEJ037 study was a multicenter prospective observational study that included patients with advanced lung cancer. In the Rising-VTE/NEJ037 study, the Khorana score was calculated for enrolled patients with available data on all Khorana score components. The modified Khorana score was based on the body mass index of ≥ 25 kg/m^2^, according to the Japanese obesity standard. A multivariate logistic regression analysis, including patient background characteristics, was performed to evaluate the presence of VTE 2 years after the lung cancer diagnosis.

**Results:**

This study included 1008 patients with lung cancer, of whom 100 (9.9%) developed VTE. From the receiver operating characteristic curve analysis, VTE risk could not be determined because both the Khorana score (0.518) and modified Khorana score (0.516) showed very low areas under the curve. The risk factors for VTE in the multivariate analysis included female sex, adenocarcinoma, performance status, N factor, lymphocyte count, platelet count, prothrombin fragment 1 + 2 and diastolic blood pressure.

**Conclusion:**

The Khorana score, which is widely used in cancer-VTE risk assessment, was less useful for Japanese patients with advanced lung cancer. Prothrombin fragment 1 + 2, a serum marker involved in coagulation, was more suitable for risk identification.

**Clinical trial information:**

jRCTs061180025.

## Introduction

Venous thromboembolism (VTE) is one of the most common medical complications during cancer treatment, and its risk in patients with lung cancer is especially high [1]. Chemotherapy increases VTE risk [2, 3], and with the advancements in cancer chemotherapy, the long-term survival of patients with lung cancer is now plausible [4]. Therefore, it is increasingly important to manage complications such as VTE, and oncologists need to be mindful of its co-development when treating lung cancer patients.

The National Comprehensive Cancer Network and American Society of Clinical Oncology have published guidelines for managing cancer-associated VTE [5, 6]. Hospitalized patients admitted for purposes other than induction of short-term chemotherapy, who show no contraindications for anticoagulant therapy, should receive treatment with unfractionated heparin, low molecular weight heparin, or direct oral anticoagulants (DOACs) for preventing VTE. Furthermore, numerous risk score tools have been proposed to evaluate cancer-associated VTE [7, 8]; however, the Khorana score [7] is the most commonly used risk assessment tool for patients who are scheduled to receive chemotherapy. To prevent VTE in patients with lung cancer receiving outpatient treatments, the Khorana score should be used to evaluate VTE risk, and patients with intermediate VTE risk (Khorana scores of ≥ 2 points) should receive similar drugs as inpatients. This recommendation follows evidence of the efficacy of prophylactic administration of DOACs in two placebo-controlled trials [9, 10] conducted to determine the prophylactic effect of DOACs on VTE in patients with cancer (with Khorana scores ≥ 2 points) who were scheduled to undergo chemotherapy.

The Khorana score incorporates five clinical and laboratory risk factors: type of cancer, platelet count, hemoglobin level and use of erythropoiesis-stimulating agents, white blood cell (WBC) count, and body mass index (BMI), and lung cancer is considered to be high-risk. van Es et al. [11] evaluated the clinical utility of multiple risk scores (Khorana, PROTECHT, and CONKO scores) to predict VTE risk in a prospective cohort of 260 patients with various cancers, including lung cancer, scheduled to undergo chemotherapy. Their findings suggest that the existing risk assessment scores may be inadequate; thus, a new risk score is required. Three retrospective validation studies of the Khorana score examining only lung cancer cases have reported that the score is not very useful for assessing VTE risks [12–14]. A meta-analysis involving multiple cancers other than lung cancer reported that the Khorana score performance for lung cancer differed from that for other cancers and that it was not useful in predicting VTE in lung cancer [15]. Furthermore, the Khorana score criterion for obesity is a BMI of ≥ 35 kg/m^2^; however, validations were also performed for a modified Khorana score based on a BMI of ≥ 25 kg/m^2^ in an Asian population [13, 14]. Optimal management of complications such as VTE can maximize the effectiveness of highly personalized lung cancer treatments; hence, there is an urgent need to validate the Khorana score using prospective observational studies data.

The Rising-VTE/NEJ037 study, a physician-led, multicenter, prospective, observational study, determined the incidence rate of VTE and its risk factors while treating lung cancer patients for whom radical treatments were unsuitable [16]. To the best of our knowledge, the Rising-VTE/NEJ037 study is the largest prospective study involving intensive screening for VTE at the time of lung cancer diagnosis, along with a further follow-up, to assess VTE incidence. Since many cases of VTE co-developing with lung cancer are asymptomatic, an appropriate risk assessment score system is essential for identifying patients who should undergo aggressive screening and monitoring.

Here, we report the results of a validation study of the Khorana score and VTE risk, including gene mutations, such as epidermal growth factor receptor (*EGFR*) gene mutation status and anaplastic lymphoma kinase (*ALK*) fusion gene, using data from a prospective observational study of advanced lung cancer (the Rising-VTE/NEJ037 study).

## Materials and methods

### Patients

Case enrollment for the Rising-VTE/NEJ037 study was conducted between June 2016 and August 2018, and patients were followed up for 2 years until August 2020. The main eligibility criteria were: the diagnosis of small cell lung cancer (SCLC) or non-SCLC (NSCLC) based on cytological or histological examinations; an Eastern Co-operative Oncology Group performance status (PS) of 0–3; patients aged ≥ 20 years when consenting; SCLC for which radical surgery, radiotherapy, and chemotherapy were impossible (regardless of categorization of SCLC as a limited or extensive disease); NSCLC for which radical surgery, radiotherapy, and chemotherapy were impossible (regardless of disease stage); postoperative or disease recurrence after radical radiotherapy; patients’ conditions were contraindicated for aggressive treatments such as chemotherapy; and expected survival period of > 6 months after consent.

Since the Rising-VTE/NEJ037 study was observational, no exclusion criteria were set for case enrollment.

Patients who met the Rising-VTE/NEJ037 study eligibility criteria underwent evaluations for VTE co-development by contrast-enhanced computed tomography (CT) scans of the chest to the lower extremities or of the chest to the pelvic CT, along with lower-extremity venous ultrasound. Furthermore, patients were classified into either the observation group without VTE co-development or the cancer-associated VTE group [16]. VTE diagnoses were confirmed through a central review by two radiologists. The following evaluations of patients with deep vein thrombosis (DVT) and pulmonary thromboembolism were performed to diagnose VTE.

### Validation for Khorana and modified Khorana scores

The Khorana score was calculated for the Rising-VTE/NEJ037 study enrolled patients who had available data on its components (platelet count, hemoglobin level, use of erythropoiesis-stimulating agents, WBC count and BMI) by assigning one point for each component. The modified Khorana score used a BMI of ≥ 25 kg/m^2^ according to the Japanese obesity standard [17]. Since all the registered Rising-VTE/NEJ037 study patients had lung cancer, one point was assigned for the primary site; therefore, the score could range from 1 to 5 points. In both the original and modified Khorana scores, the risk scores were categorized as intermediate-low risk, intermediate-high risk, and high risk for 1, 2 or ≥ 3 points, respectively.

### Risk assessment for VTE

To identify VTE risk, we included patients with complete background information and clinical data, including hematological examinations and imaging data that could be used to confirm the presence of VTE. The parameters used for risk assessment included age, sex, BMI, histological classification of cancer, TNM factor, PS score, past medical history (stroke, myocardial infarction, and other malignant tumors), comorbidities (chronic obstructive pulmonary disease, rheumatoid arthritis, diabetes, hypertension, dyslipidemia and other malignant tumors), complete blood cell count, coagulation markers (d-dimer, prothrombin fragment 1 + 2 [PT F1 + 2]), liver function markers, kidney function markers, electrolyte levels, C-reactive protein (CRP) levels, brain natriuretic peptide (BNP) levels, oxygen saturation (SpO_2_), blood pressure, *EGFR* gene mutation status and *ALK* fusion gene. All clinical data were available during the lung cancer diagnosis.

### Statistical analyses

The target sample size of the Rising-VTE/NEJ037 study was aimed to exceed those of large-scale cohorts reported so far because the incidence of VTE complication rate in Japanese patients with lung cancer was unknown at the time the study was planned. Since the prospective cohort trial at that time was on a scale of hundreds of cases, the target sample size for this trial was set to 1000.

After performing the univariate logistic regression analysis for each factor, we performed a multivariate logistic regression analysis using a stepwise method to identify VTE risk factors. Variables that were significant in the univariate analysis were employed in the multivariate model, and variables with multicollinearity problems were checked and excluded in a prior correlation analysis. To validate the Khorana and modified Khorana scores, we plotted the receiver operating characteristic (ROC) curves for each continuous variable included in the multivariate analysis and calculated their sensitivity and specificity. The ROC analysis was performed to estimate the respective cut-off values for each item to be set in the scoring process. All statistical analyses were conducted using IBM SPSS Statistics for Windows, version 24.0 (IBM Japan, Ltd., Tokyo, Japan).

### Ethics

This study was conducted in accordance with the principles of the Declaration of Helsinki and the Good Clinical Practice Guidelines. The study protocol was approved by the Shimane University Institutional Review Board based on the Clinical Trials Act enacted in Japan in 2017 and published in the Japan Registry of Clinical Trials (jRCTs061180025). Written informed consent was obtained from the patients.

## Results

### Patient characteristics and VTE incidence rate

The Rising-VTE/NEJ037 study included 1021 patients diagnosed with lung cancer unsuitable for radical resection or radiation between June 2016 and August 2018 across 35 institutions in Japan. After excluding 13 patients with missing radiological data, the remaining 1008 patients constituted the full analysis set.

The enrolled patients’ median age was 70 (range 30–94) years, and most were males (714 patients, 70.8%) and had good PS (0–1, 80.6%). The most common histological lung cancer subtypes were adenocarcinoma (641 patients, 63.6%). The disease stage was assessed according to the 7th edition TNM staging system for lung cancer (UICC-7) [18]; the M1a,b stage IV disease accounted for 80% of all cases [16]. Overall, 62 and 38 patients had VTE at lung cancer diagnosis and over the two years of follow-up, respectively, totaling 100 VTE cases.

### Validation of the Khorana and modified Khorana scores

**Fig. 1:**
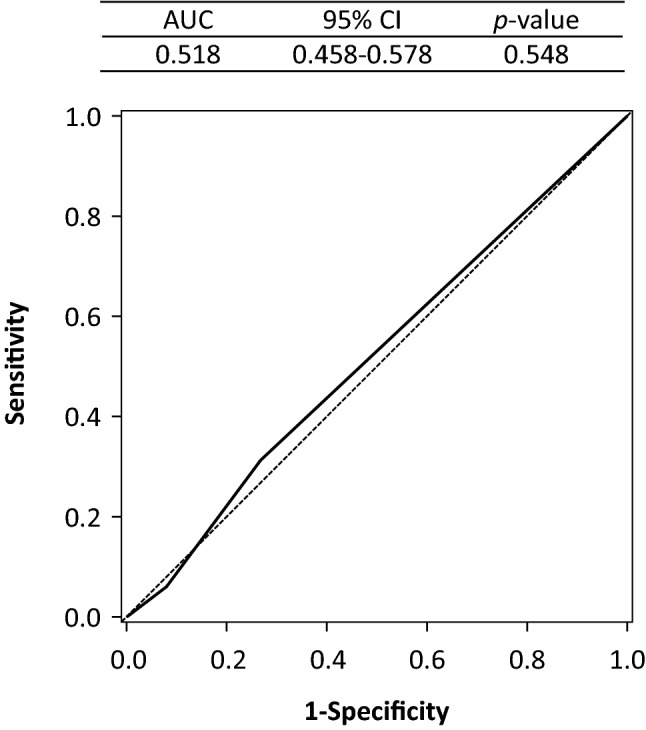
Evaluation of the discriminating ability of Khorana score for venous thromboembolism (receiver operating characteristic curve). *AUC* area under the curve, *CI* confidence interval

The Khorana score was calculated for 1003 patients enrolled in the Rising-VTE/NEJ037 study, and VTE co-development over the two-year observation period was confirmed in 99 patients (9.9%). According to the Khorana score, 925 (92.2%) patients were categorized as at intermediate risk of VTE development, including 730 (72.8%) and 78 (7.8%) categorized as intermediate-low (1 point) and high risk, respectively, indicating that most patients were in the intermediate risk category (Table 1A). VTE incidence rates were 9.3, 12.8 and 7.6% in the intermediate-low risk (1 point), intermediate-high risk (2 points), and high-risk categories, respectively. However, the sensitivity was low at 0.061, and ROC analysis demonstrated a very low area under the curve (AUC 0.518; 95% CI 0.458–0.578; *p* = 0.548), suggesting that the Khorana score could not discriminate VTE risks (Fig. 1).

**Table 1:** Presence or absence of VTE co-development by Khorana score

	Khorana score			Total
	IM-L (1 point)	IM-H (2 points)	H (3–5 points)	
(A) Khorana score				
No VTE (N)	662	170	72	904
VTE presence (N)	68	25	6	99
VTE incidence rate	9.3%	12.8%	7.6%	9.9%
(B) Modified Khorana score				
No VTE (N)	524	292	88	904
VTE presence (N)	53	38	8	99
VTE incidence rate	9.2%	11.5%	8.3%	9.9%

The sensitivity and specificity of the Khorana score for the risk prediction of VTE co-developing with lung cancer were 0.061 and 0.920, respectively. The sensitivity and specificity of the modified Khorana score for the risk prediction of VTE co-developing with lung cancer were 0.081 and 0.903, respectively

*IM-L* intermediate-low, *IM-H* intermediate-high, *H* high, *VTE* venous thromboembolism

**Fig. 2:**
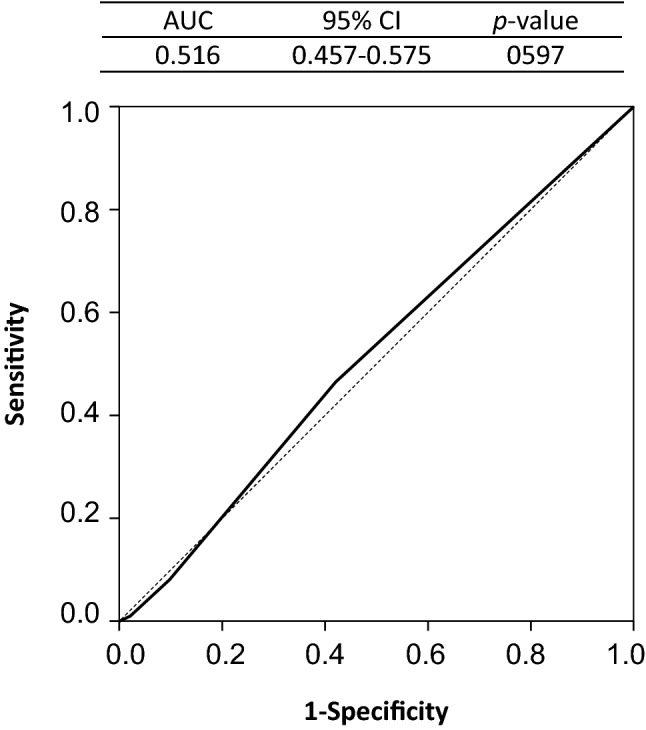
Evaluation of the discriminating ability of modified Khorana score for venous thromboembolism (receiver operating characteristic curve). *AUC* area under the curve, *CI* confidence interval

According to the modified Khorana score, 907 (90.4%) patients were categorized as at intermediate risk of VTE development, including 577 (57.5%) and 96 (9.6%) categorized as intermediate-low (1 point) and high risk, respectively, indicating that most patients were in the intermediate risk category (Table 1B). VTE incidence rates were 9.2, 11.5 and 8.3% in the intermediate-low risk (1 point), intermediate-high risk (2 points), and high-risk categories, respectively. However, the sensitivity was low at 0.081 and ROC analysis demonstrated a very low AUC (0.516; 95% CI 0.457–0.575; *p* = 0.597), suggesting that the modified Khorana score could not discriminate VTE risks (Fig. 2).

### Identification of the risk factors for VTE

After the univariate analysis (Table 2) including the patient background data (age, sex, BMI, PS, medical history, and comorbidities); tumor-related factors (histological classification, TNM factors, *EGFR* gene mutation and *ALK* fusion gene); and physiological examinations and blood biochemistry tests results (complete blood cell count, coagulation markers, liver function, kidney function, electrolytes, CRP, BNP, SpO_2_ and blood pressure); the multivariate analysis showed eight factors (female sex, adenocarcinoma, N3, poor PS, low lymphocyte count, low platelet count, high PT F1 + 2 and high diastolic blood pressure [DBP]) as risk factors of VTE [32].

## Discussion

The Rising-VTE/NEJ037 study is the largest prospective study to intensively screen patients for VTE at lung cancer diagnosis, monitor their subsequent progression, and determine the incidence rate of VTE [16]. The rate of VTE co-development over 2 years of follow-up was 9.9%. We present the first study to use a large prospective, observational cohort data to assess VTE risk and validate the ineffectiveness of the Khorana score in patients with advanced lung cancers.

Multiple retrospective studies have reported that VTE development is common in women [19, 20]. A retrospective study [13] including Japanese patients with lung cancer suggested that VTE co-development is common in adenocarcinoma cases, and both claims are consistent with this study’s results. By contrast, while the Khorana score [9] suggests that a high platelet count increases VTE risk, our results suggest that a greater VTE risk development was associated with a low platelet count. The results of a similar prospective observational study of Japanese patients with advanced cancers [21] also indicated a low platelet count as a risk factor for VTE development. Although it has been speculated that this finding is because of the higher platelet consumption levels and subsequent tendency toward thrombus formation in the body, it is yet to be verified. The possibility that low lymphocyte counts are a risk factor for VTE was also reported for the first time in the Rising-VTE/NEJ037 study and requires future verification. Regarding the relationship between blood pressure and VTE, Mahmoodi et al. examined the risk factors for cardiovascular diseases and their association with VTE in a meta-analysis of nine clinical trials [22]. They reported that the VTE onset was inversely associated with systolic blood pressure (SBP) but not with DBP. The Rising-VTE/NEJ037 study results indicated an association between VTE incidence and elevated DBP but not with SBP. Thus, additional investigations are required to confirm whether the risk factors for cardiovascular diseases are associated with VTE.

Additionally, d-dimer was included in the Vienna VTE risk score model as a negative serum marker of VTE, and a d-dimer level of ≥ 1.44 mg/mL indicated VTE risk development [23]. Adjustment of the d-dimer cut-off level was shown to increase the Khorana score sensitivity in a prospective cohort study [24]. Here, an elevated d-dimer level was not a risk factor, and PT F1 + 2, as a serum marker involved in coagulation, was more suitable for risk identification (Table 3). d-dimer refers to the degradation products of a thrombus, a stabilized fibrin by plasmin, and it reflects the formation of fibrin in vivo only. Since PT F1 + 2 is released when activated factor X cleaves prothrombin into thrombin, its elevation indicates early-stage thrombus formation. PT F1 + 2 was reported to be particularly useful as a predictor of cancer-associated thrombosis (CAT) when used in combination with d-dimer [25], and it is necessary to reconfirm its usefulness and further consider its utilization in future studies.

**Table 2:** Univariate analysis of VTE risk

Parameter	OR	95% CI	*p* value
Sex, (F vs. M)	1.975	1.295–3.011	0.002
Histological type, (small vs. non-small)	0.379	0.162–0.882	0.024
Adenocarcinoma vs. NSCLC others	2.173	1.329–3.553	0.002
Squamous cell carcinoma vs. NSCLC others	0.630	0.344–1.155	0.135
Large cell carcinoma vs. NSCLC others	n.c.		
NSCLC_STAGE			
Ib	n.c		
IIa	n.c		
IIb	n.c		
IIIa	0.386	0.048–3.094	0.370
IIIb	0.446	0.122–1.636	0.224
IV	1.491	0.788–2.822	0.220
Postoperative recurrence	1.000	ref	
T factor			
T1a	1.000	ref	
T1b	0.418	0.133–1.317	0.136
T2a	0.497	0.169–1.458	0.203
T2b	0.920	0.282–2.999	0.890
T3	0.451	0.154–1.321	0.146
T4	0.439	0.154–1.255	0.124
TX	0.558	0.135–2.303	0.420
N factor			
0	1.000	ref	
1	1.542	0.627–3.795	0.346
2	1.297	0.622–2.703	0.488
3	2.316	1.207–4.443	0.012
ECOG PS			
0	1.000	ref	
1	2.070	1.271–3.371	0.003
2	1.833	0.793–4.237	0.157
3	3.665	1.533–8.767	0.004
*EGFR* mutation positive	4.572	1.376–15.189	0.013
*ALK* fusion gene positive	5.732	1.763–18.635	0.004
Comorbidity			
COPD	0.769	0.446–1.327	0.345
Rheumatoid arthritis	1.524	0.336–6.908	0.585
Diabetes metritis	0.500	0.262–0.955	0.036
Malignant tumor	0.391	0.094–1.638	0.199
Hypertension	0.861	0.564–1.314	0.487
Dyslipidemia	0.879	0.526–1.471	0.624
Other	0.764	0.492–1.188	0.232
Medical history			
Stroke	0.918	0.386–2.185	0.848
Myocardial infarction	2.120	0.785–5.726	0.138
Malignant tumor	0.620	0.315–1.221	0.167
Other	0.807	0.515–1.264	0.349
AGE (per 1)	0.992	0.972–1.013	0.468
WBC (per 1/μL)	1.000	1.000–1.000	0.976
NEUT (per 1/µL)	1.000	1.000–1.000	0.554
EOS (per 1%)	0.932	0.849–1.024	0.142
BASO (per 1%)	0.561	0.304–1.035	0.064
MONO (per 1%)	0.954	0.867–1.050	0.339
LYMPH (per 1%)	0.969	0.946–0.993	0.011
Hemoglobin (per 1 g/dL)	0.972	0.861–1.097	0.643
PLT (per 10,000/μL)	0.975	0.953–0.997	0.027
PT-INR (per 1 s)	0.890	0.260–3.046	0.853
APTT (per 1%)	0.952	0.906–1.000	0.050
d-dimer (per 5 μg/mL)	1.232	1.111–1.365	0.000
PT F1 + 2 (per 50 pmol/L)	1.120	1.073–1.169	0.000
Total protein (per 1 g/dL)	0.697	0.495–0.982	0.039
ALB (per 1 g/dL)	0.859	0.603–1.224	0.400
LDH (per 1U/L)	1.000	1.000–1.001	0.273
AST (per 1U/L)	1.001	0.992–1.010	0.858
ALT (per 1U/L)	1.001	0.991–1.011	0.855
BUN (per 1 mg/dL)	0.979	0.937–1.023	0.343
Crea (per 1 mg/dL)	0.352	0.112–1.110	0.075
T-Bil (per 1 mg/dL)	0.884	0.521–1.500	0.648
NA (per 1 mEq/L)	0.998	0.940–1.059	0.950
K (per 1 mEq/L)	1.099	0.668–1.810	0.710
CL (per 1 mEq/L)	1.010	0.956–1.066	0.729
CRP (per 1 mg/dL)	1.001	0.954–1.049	0.978
BNP (per 1 pg/mL)	1.001	0.999–1.003	0.187
CCR (per 1 mL/min)	1.003	0.995–1.011	0.481
SPO_2_ (per 1%)	0.892	0.804–0.988	0.029
sBP (per 1 mmHg)	1.008	0.995–1.020	0.235
dBP (per 1 mmHg)	1.019	1.000–1.038	0.049

*VTE* venous thromboembolism, *OR* Odds ratio, *95% CI* 95% confidence interval, *ref* Reference, *n.c*. incalculable

**Table 3:** Distribution of prothrombin fragment 1 + 2

All *N* = 1008	All *N* = 1008	With VTE *N* = 62	Without VTE *N* = 946	*p *value
Prothrombin fragment 1 + 2				
Median	289	442	284	< 0.001
IQR	207, 414	307, 670	204, 403	
Range	15, 1201	15, 1201	28, 1201	

*p* value was calculated using the Mann–Whitney *U* test

With the increasing personalization of chemotherapy for lung cancer, patients are undergoing testing for gene mutations, and treatments are being tailored according to such test results [26]. Many researchers are interested in identifying the relationship between the presence or absence of gene mutations and VTE onset. A retrospective study of patients with *ALK* fusion gene-positive lung cancer demonstrated a significantly higher VTE incidence among 422 patients with *ALK* fusion gene-positive lung cancer than that in patients who were negative for the fusion gene (42.7% vs. 28.6%, *p* < 0.0001) [27]. Furthermore, a prospective cohort study involving 341 patients with lung cancer, including 26 positive for *ALK* fusion gene, indicated a higher cumulative VTE incidence rate of 26.9% in *ALK* fusion gene-positive patients (9.2% in *ALK* fusion gene-negative patients) [28]. In assessing other gene mutations (*EGFR* and *KRAS*), a meta-analysis of 25 studies suggested that neither *EGFR* nor *KRAS* gene mutations is risk factors for VTE development [29]. We only studied whether *EGFR* gene mutation and *ALK* fusion gene are risk factors for VTE and found that neither was a risk factor. This discrepancy may be due to differences in the patient populations studied. Hence, the Rising-VTE/NEJ037 study evaluated patients with advanced lung cancer, with SCLC, and with highly-differentiated squamous cell carcinoma who did not undergo genetic testing in routine clinical practice. It is necessary to conduct a prospective observational study involving only those patients positive for these gene mutations to examine the link between each gene mutation and VTE development.

This study had some limitations. First, the study was limited to Japanese patients with advanced lung cancers. Despite a 9.9% VTE incidence rate of VTE that is comparable to the reported VTE incidence rates requiring treatments in the US and Europe, many hereditary coagulation factor abnormalities associated with thrombosis show racial differences [30]. Thus, it is necessary to examine whether the results of this study are similar to those of patients in other Asian countries. Second, PT F1 + 2, which was identified as a risk factor in the present study, has not been measured in routine clinical practice or clinical trials on CAT. Therefore, it is necessary to design a new study to validate its usefulness as a risk factor. Third, the chemotherapy-induced increase in VTE risk could not be examined because > 80% of patients in the study were receiving chemotherapy. In particular, because existing data indicate an increased risk of developing VTE due to the use of angiogenesis inhibitors [31], studies to evaluate risk based on the presence or absence of chemotherapy and administered drug type will be desirable in the future.

VTE incidence during cancer treatment can be an important factor affecting patients’ vital prognosis. In routine clinical practice, a comprehensive evaluation of VTE risk development based on each patient’s clinical information and appropriate treatment selection is crucial. This is the first study to evaluate the usefulness of the Khorana and modified Khorana scores for identifying VTE development in patients with lung cancer, using data from a large-scale prospective, observational study. The findings suggest that changing the BMI in the Khorana score may not be useful for evaluating VTE risk in Japanese patients with lung cancer. From the Rising-VTE/NEJ037 dataset, we are proposing a new risk assessment scoring system for VTE development [32]. The clinical utility of a new risk assessment scoring system relevant to the pre-chemotherapy assessment of patients with advanced lung cancer needs to be confirmed in future studies. As the number of cancer patients achieving long-term survival increases, we hope that in the future, more emphasis will be placed on the diagnosis and treatment of VTE co-occurring with cancer.
